# Functional Polymorphisms and Gene Expression of TLR9 Gene as Protective Factors for Nasopharyngeal Carcinoma Severity and Progression

**DOI:** 10.1155/2019/2826563

**Published:** 2019-11-26

**Authors:** Macherki Yosra, Souissi Sameh, Ghedira Randa, Remadi Yassmine, Gabbouj Sallouha, Bouzid Nadia, Bouaouina Noureddine, Zakhama Abdelfattah, Hassen Elham

**Affiliations:** ^1^Laboratory of Molecular Immuno-Oncology, Faculty of Medicine of Monastir, Monastir University, 5019 Monastir, Tunisia; ^2^Higher Institute of Biotechnology of Monastir, Monastir University, Monastir, Tunisia; ^3^Department of Cancerology and Radiotherapy, Farhat Hached University Hospital, Sousse University, Sousse, Tunisia; ^4^Department of Anatomy and Pathologic Cytology, Fattouma Bourguiba University Hospital, Monastir University, Monastir, Tunisia

## Abstract

Nasopharyngeal carcinoma (NPC) is a disease that is closely associated with EBV infection. Toll-like receptor 9 is an important factor mediating the interaction between EBV and the host immune response. Any genetic (single nucleotide polymorphisms, SNPs) or expression variation in TLR9 gene may modify the ability of the receptor to respond correctly to viral infection as in NPC. This study is aimed at evaluating the effect of TLR9 functional polymorphisms (TLR9-1486 T/C and TLR9-1237 T/C) and TLR9 mRNA expression in NPC severity and progression at diagnosis and after treatment. This study included 322 patients with NPC. RFLP-PCR and real-time PCR were used to assess, respectively, the genotypes and the mRNA expression of TLR9 gene. The genotyping analysis showed that the presence of mutated allele -1237C (TLR9-1237 TC+CC) was associated with large tumor size (*p* = 0.017; OR (CI 95%) = 1.888 (1.11-3.19)) at diagnosis. After treatment, the -1237C allele was associated with a better chance of complete remission (*p* = 0.031, OR (CI 95%) = 0.486 (0.25-0.95)), a lower risk of distant metastasis (*p* = 0.028, OR (CI 95%) = 0.435 (0.18-1.02)), and a lower risk of death by NPC (*p* = 0.003, OR (CI 95%) = 0.20 (0.06-0.67)). Kaplan-Meier analysis showed that patients with -1237CC and -1237TC genotypes had a better overall survival (OVS) (*p* < 0.01) and distant metastasis-free survival (DMFS) (*p* < 0.05). A multivariate analysis revealed that TLR9-1237 T/C polymorphism was an independent prognostic factor in OVS (*p* = 0.02; HR = 0.244) and DMFS (*p* = 0.048; HR = 0.388). The transcriptomic analysis showed that the mRNA expression was reduced in patients with larger tumor size (T4) (*p* = 0.013) and advanced clinical stage (SIII-SIV) (*p* = 0.037). The TLR9 mRNA expression was inversely correlated with tumor size (*p* = 0.014; *r* = −0.314) at diagnosis. Our results indicated for the first time that the functional -1237 T/C polymorphism and mRNA expression of TLR9 gene may be considered as protective factors for NPC severity and progression.

## 1. Introduction

Nasopharyngeal carcinoma (NPC) is a tumor derived from the epithelial cells and usually occurs in the fossa of Rosenmüller. NPC is a distinct entity compared to other epithelial cancers of the head and neck regions in its radiosensitivity, close association with EBV infection, and remarkable geographic distributions.

While it is rare in most parts of the world such as Europe and North America, this disease is the major cause of cancer death in China, particularly among people of Cantonese origin where the incidence exceeds 25 per 100000 (man/year) (26.6/100000 in Guangdong, 15.7/100.000 in Maca, and 14.4/100000 in Hong Kong) [1]. Intermediate incidence rates were recorded in Southeast Asia and in North Africa, principally in Tunisia, Algeria, and Morocco, where the incidence rates reached 2-5 per 100000 inhabitants [2, 3]. The unequal geographic distribution in NPC incidence suggests that NPC is a multifactorial disease which results from the combined action of multiple etiological factors such as lifestyle, EBV infection, and genetic factors.

Various environmental factors such as salt-preserved food intake, tobacco consumption, and fume inhalation increase the risk of developing NPC [4, 5]. Likewise, exposure to Epstein-Barr virus has been consistently linked with the risk of developing NPC. In our previous study, we have demonstrated that in untreated NPC patients the EBV viral load was approximately 100 times higher compared with healthy controls [6]. More recently, it has been shown that high plasma EBV-DNA copies decrease the survival rates of patients with nasopharyngeal cancer [7]. During viral infection, the innate immune system is our first line of defense that organizes host responses to prevent the replication of the virus. The family of Toll-like receptors (TLRs) is an important factor mediating the interaction between viral agents and the host immune response. The TLRs are transmembrane pattern-recognition receptors (PRRs) that mediate innate immune responses when exposed to pathogen-associated molecular patterns (PAMPs) such as bacterial lipopolysaccharide, microbial RNA, or DNA [8]. So far, 13 TLRs have been identified in mammals and 10 in humans [9].

The human TLR9 is a DNA receptor that recognizes unmethylated nucleic acid containing Cytosine-phosphate-Guanine (CpG) motifs present in bacteria and viruses [10]. In the innate immune system, the single nucleotide polymorphisms (SNPs) within the TLR9 gene may alter the ability of the receptor to respond correctly to TLR9 ligands. They also participate in the physiopathology of many inflammatory and immune diseases, including cancer [11]. The SNP may influence gene expression, mRNA stability, protein function, and subcellular localization of mRNAs and/or proteins [12]. In the last years, several genetic association studies have demonstrated the impact of TLR9 polymorphisms on several cancers, including lymphoma, Hodgkin's lymphoma, endometrial cancer, gastric cancer, non-Hodgkin's lymphoma, cervical cancer, acute myeloid leukemia, breast cancer, and colorectal cancer [13–21].

Two functional SNPs (TLR9-1486 T/C (rs187084) and TLR9-1237 T/C (rs5743836)) are located in the promoter. They modify the transcriptional activity and the gene expression [22, 23]. The aim of the present study was to investigate firstly the effect of TLR9 promoter polymorphism on NPC pathogenesis at diagnosis and after treatment and their impact on survival rates. Secondly, the study was aimed at evaluating the expression levels of TLR9 mRNA in peripheral blood mononuclear cells (PBMCs) of NPC patients and their association with the NPC pathogenesis.

## 2. Materials and Methods

### 2.1. Study Population

This study included 322 patients with NPC. They were recruited from the Department of Radiation Oncology and Medical Oncology at Farhat Hached University Hospital. The study was approved by the National Ethical Committee, and an informed consent was obtained from all enrolled individuals prior to their participation. Age varied from 10 to 85 years, with a mean age of 42.62 ± 15.46 (Table 1). The distribution of age-specific rates for NPC patients showed a bimodal age distribution, with the first peak between 10 and 22 and the second one around the age of fifty. Respecting the distribution bimodal of age in intermediate risk regions, young patients were defined as those aged 35 years or under while adult patients were over 35. The sex ratio was 2.19 (219 men, 100 women). The diagnosis of NPC was confirmed by a histopathological analysis. In Tunisian NPC patients, the UCNT (undifferentiated carcinoma, type III, WHO classification) is the most predominant type in the histological repartition (89.6%) compared with differentiated types (only 5.3%) (type I, II, WHO classification) [24]. In accordance with the 1997 TNM staging of malignant tumors, the NPC clinical stages ranged from I to IV [25]. In the present study, all NPC patients were diagnosed with UCNT with a lymph node extension N+ (77.6%), advanced clinical stage III, IV (91.3%) (Table 1).

At the time of this study, 215 patients were in complete remission and 79 patients relapsed after treatment. Among these, 32 patients developed locoregional recurrence, 31 patients developed distant metastasis, and 13 patients had both types of relapse after treatments. In our population, nearly half of the patients who relapsed early died of nasopharyngeal cancer (39 patients, 49.4%) (Table 1).

### 2.2. Genomic DNA Extraction and TLR9 Genotyping Analysis

Peripheral blood was collected in EDTA tubes. After centrifugation, genomic DNA was extracted from the blood sample by a salting out method [26, 27].

TLR9 promoter polymorphisms (TLR9-1486 T/C (rs187084) and TLR9-1237 T/C (rs5743836)) were genotyped using the restriction fragment length polymorphism of polymerase chain reaction (RFLP-PCR) analysis. All PCR reactions were performed in a 25 *μ*l reaction volume containing 100 ng of genomic DNA, 200 *μ*M dNTP, 1x Taq polymerase buffer with MgCl_2_, unit of Taq DNA polymerase (Promega, Paris, France), and 0.3 *μ*M of suitable primers described previously [28, 29]. For the genotyping of TLR9-1486 T/C polymorphism, 145 pb was amplified after 35 cycles of PCR with the following temperature program: denaturation at 95°C for 30 seconds, annealing primers at 62°C for 30 seconds, and primer extension at 72°C for 30 seconds. For TLR9-1237 T/C polymorphism, 154 pb was amplified using the following conditions, thermal cycler (Biometra, Göttingen, Germany): 95°C for 5 min followed by 30 cycles (95°C for 30 seconds, 60°C for 30 seconds, and 72°C for 30 seconds) and a final elongation step carried out at 72°C for 5 min.

After amplification, the PCR products were digested at 37°C overnight with 4 U of AflII restriction enzyme (Thermo Scientific) for the −1486 polymorphism and with 2 U of BstNI restriction enzyme (Thermo Scientific) for the −1237 polymorphism. The digestion products were then separated by a 3% agarose gel stained with ethidium bromide and visualized with ultraviolet light. After electrophoresis, for the -1486 T/C polymorphism, the -1486T allele was detected by the presence of 111 and 34 bp bands, whereas the -1486C allele was defined by the loss of the AflII site, yielding an undigested 145 bp fragment. For -1237 T/C, homozygous T alleles were represented by DNA bands with sizes at 129 and 25 bp. The presence of 81, 48, and 25 pb size fragments designated the homozygous C alleles while the heterozygous genotype displayed a combination of both alleles (129, 81, 48, and 25 bp). The band 25 pb indicated the digestion in the constant restriction site. All restricted fragments were analyzed by electrophoresis on a 3% agarose.

### 2.3. Quantitative Real-Time Reverse Transcription PCR (Real-Time RT-PCR) for TLR9 Gene Expression

Quantitative real-time PCR (qPCR) was used to evaluate TLR9 mRNA expression in the PBMCs. Peripheral blood samples were collected from patients in EDTA vacutainer tubes, and the PBMCs were isolated using the Ficoll density gradient technique. The total RNA was extracted from PBMC using the RNeasy Mini Kit (Qiagen, Hilden, Germany) following the manufacturer's instructions. Using a NanoDrop 2000c Spectrophotometer, RNA concentration was assessed by measuring absorption at 260 nm and the purity was evaluated by measuring the 260/280 nm ratio. The integrity of the total RNA was then checked on 1% agarose gel by the presence of ribosomal RNA (rRNA) bands (28S and 18S). One hundred microgram of total RNA was reverse transcribed into cDNA by the use of the first-strand cDNA synthesis kit (Fermentas, St. Leon-Rot, Germany) which uses random Hexamer and Oligo (Dt) primers. For real-time PCR, 2 microliters of the cDNA was used and the reaction was carried out using iQ™ SYBR® Green Supermix (Bio-Rad, CA) in an iQ5 Multicolor Real-Time PCR Detection System (Bio-Rad, Hercules, CA). The cycling conditions were initiated by a denaturation step at 95°C for 10 min which was followed by 40 cycles at 95°C for 15 s and 58.2°C for 1 min. For normalization, the housekeeping GAPDH gene was used for normalization as an internal control. The relative expression of TLR9 gene was determined using the 2^−*ΔΔ*CT^ method. The PCR primers used in TLR9 quantification were described previously [30, 31].

### 2.4. Statistical Analysis

The chi-square test (*χ*^2^) was used to evaluate any significant association between clinicopathological features (age “≤35 years versus >35 years,” sex “men versus women,” primary tumor extension “T1-T2 versus T3-T4,” regional lymph node extension “N0 versus N+,” metastatic status “M0 versus M+,” and clinical stages “I-II versus III-IV”) and TLR9 polymorphisms. In the present study, we investigated the association between TLR9 promoter polymorphisms and responses after treatment: recurrence after treatment “No versus Yes,” locoregional recurrence after treatment “No versus Yes,” distant metastasis after treatment “No versus Yes,” and death after treatment and after recurrence “No versus Yes.” Fisher's exact test was appropriate when the sample sizes were small (sample is less than 5). Odds ratios (OR) and 95% confidence intervals (CI) were calculated to estimate the relative risk.

Nasopharyngeal carcinoma-specific overall survival (OVS) was defined as the time from the date of diagnosis to death if the patient died from nasopharyngeal carcinoma or to the last contact. Distant metastasis-free survival (DMFS) was defined as the time from the date of diagnosis to the first metastasis or to the last contact, and (LRRFS) locoregional relapse-free survival was defined as the time from the date of diagnosis to the first locoregional relapses or to the last contact. Survival rates were estimated using the Kaplan-Meier method for calculating survival curves. The differences between groups were calculated by the log-rank test. Univariate and multivariate analyses were carried out to identify the impact of clinicopathological variables and TLR9 promoter polymorphisms on survival rates. The hazard ratio (HR) and 95% CI were calculated using the Cox regression analysis. Variables with a *p* value less than 0.01 in the univariate model and TLR9 promoter polymorphisms were included as covariates in the multivariate model.

To evaluate the role of TLR9 expression in NPC prognosis, the nonparametric Mann-Whitney *U* test wild-type was used. Spearman's rank correlation test was used to determine the direction of the relationship between the TLR9 mRNA expression levels and clinicopathological characteristics.

The data were analyzed using the Epi-Info statistical program (Version 7.1.3.10; Atlanta, USA) and a statistical software IBM SPSS for Windows (SPSS 20.0 SPSS Inc., Chicago, IL). For all experiments, a value of *p* = 0.05 was considered to indicate a statistically significant result.

## 3. Results

### 3.1. TLR9 Promoter Polymorphisms and Their Associations with NPC Clinicopathological Characteristics

The comparison of frequency distribution of TLR9 promoter genotypes with regard to demographic features of patients (sex and age) did not show any significant association (Table 2). According to the clinicopathological characteristics of NPC at diagnosis, we showed a significantly higher TLR9−1237 TC+CC distribution in the T3-T4 subgroup than in the T1-T2 subgroup. Therefore, patients with the TC+CC genotype had at the time of diagnosis a significantly larger tumor size compared to the individuals with the TT genotype (*p* = 0.017; OR (CI 95%) = 1.888 (1.11-3.19)). No significant associations were found between the other clinicopathological parameters and TLR9-1237 T/C polymorphism (Table 2). No significant associations were found between the clinicopathological parameters and TLR9-1486 T/C polymorphism either (Table 2).

In this study, we investigated the association between TLR9 promoter polymorphisms (TLR9-1486, TLR9-1237) and responses after treatment parameters including recurrence “No: complete remission versus Yes: relapse”; locoregional recurrence “No: negative locoregional recurrence versus Yes: positive locoregional recurrence”; distant metastasis “No: negative distant metastasis versus Yes: positive distant metastasis”; and death after treatment and after recurrence “No: alive versus Yes: death.”

Same significant associations were observed in the recurrence, distant metastasis, and death parameters (Table 3). Consequently, patients carrying the mutant allele -1237C (TC+CC) had a better chance of complete remission after treatment compared to those with the TT genotype (*p* = 0.031, OR (CI 95%) = 0.486 (0.25-0.95)). The same group of patients (TC+CC) was at low risk of developing distant metastasis (*p* = 0.028, OR (CI 95%) = 0.435 (0.18-1.02)) and at lowest risk of death (*p* = 0.003, OR (CI 95%) = 0.20 (0.06-0.67)) after treatment compared to patients with the TT genotype. The association was marginal between TLR9-1237 T/C and locoregional recurrence after treatment parameter (*p* = 0.051, OR (CI 95%) = 0.435 (0.18-1.02)). Unlike the TLR9-1237 T/C polymorphism findings, no significant associations were found between TLR9-1486 T/C and NPC responses after treatment (Table 3).

### 3.2. Survival Analysis and Prognostic Significance of TLR9 Promoter Polymorphisms

One of the main objectives of this study was to identify the relationship between the distribution of TLR9 promoter genotypes and the survival percentage (OVS, DMFS).

**Figure 1:**
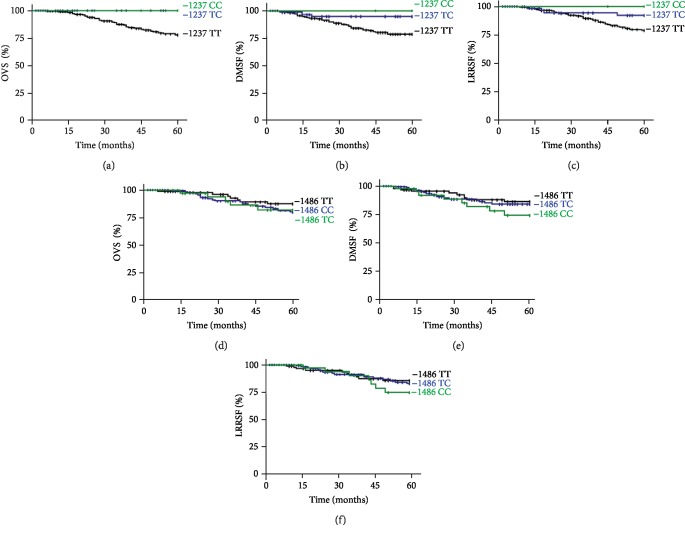
Survival analysis curves. (a, d) Overall survival (*p* < 0.01 and *p* > 0.1, respectively); (b, e) distant metastases-free survival (*p* < 0.05 and *p* > 0.1, respectively); (c, f) locoregional recurrence-free survival of NPC patients according to TLR9-1237 T/C and TLR9-1486 T/C genotypes (*p* > 0.1 and *p* > 0.1, respectively). *p* denotes the log-rank test value.

When we tested the relationship between TLR9-1237 genotypes in all the 292 patients and the survival (OVS, DMFS, and LRRFS), a significant difference was observed between the Kaplan-Meier OVS curves (*p* < 0.01) and DMFS curves (*p* < 0.05). Thus, 5 years after a cancer diagnosis, patients with CC and TC genotypes had a significantly better overall survival (100%) compared to patients with the wild-type genotype (TT) (78.2%) (Figure 1(a)). Therefore, the mean survival time was 65 and 44.2 months for patients with the variant genotypes CC and TC, respectively, and 43.5 months for those with the wild-type genotype TT (Figure 1(a)). The same significant difference was found in distant metastasis-free survival (DMFS) according to TLR9-1237 genotypes among the whole NPC patients. So, patients who were homozygous for the TLR9-1237 TT (79.4%) had a worse DMFS compared to those with other genotypes TC (92.2%) and CC (100%) (*p* < 0.05) (Figure 1(b)).

Although there was no statistical difference in LRRFS (*p* > 0.05), patients with CC and TC genotypes were at lower risk of LRRFS compared to patients carrying TT genotypes (Figure 1(c)).

Specific OVS rates were estimated and compared to previous clinicopathological parameters. Same significant associations with OVS were found for tumor size, lymph node extension, clinical stages, age subgroups, and sex (data not shown). Patients with TLR9-1237 variant genotypes (TC, CC) had a significantly better OVS compared to those with the wild-type genotype (TT). This was especially obvious in patients who had a large tumor size (*p* < 0.02), positive lymph node extension (*p* < 0.01), and advanced clinical stage (*p* < 0.01), were men (*p* < 0.05), and were older than 35 (*p* < 0.02) (data not shown). No significant differences were observed with different Kaplan-Meier survival curves (OVS, DMFS, and LRRFS) in the distribution of TLR9-1486 genotypes, among all NPC patients (Figures 1(d)–1(f)) and among the different clinicopathological parameters.

In this study, univariate and multivariate Cox regression analyses were performed to assess the effects of clinicopathological factors and the TLR9 promoter polymorphisms on OVS, DMFS, and LRRFS. The univariate analysis for OVS found prognosis to be significantly correlated to 3 clinicopathological factors at diagnosis. Therefore, we showed that age (*p* = 0.001, HR = 4.622), sex (*p* = 0.034, HR = 2.322), and metastases (*p* = 0.007, HR = 3.708) were significantly correlated with decreases of OVS. However, TLR9-1237 T/C was a significant prognostic factor for a better survival rate (*p* = 0.010, HR = 0.215) (Table 4). In a multivariate analysis, when age, metastases, and TLR9 promoter polymorphisms were included, we found that age, metastases, and TLR9-1237 T/C were independent prognostic factors (Table 4).

The multivariate analysis of DMFS showed that sex, metastasis at diagnosis, and TLR9-1237 T/C polymorphism were independent prognostic factors in DMFS (Table 4). For LRRFS, particular age was an independent prognostic factor (Table 4).

### 3.3. TLR9 Expression and Their Associations with Pathogenesis of NPC Cancer and with TLR9 Promoter Polymorphisms

**Figure 2:**
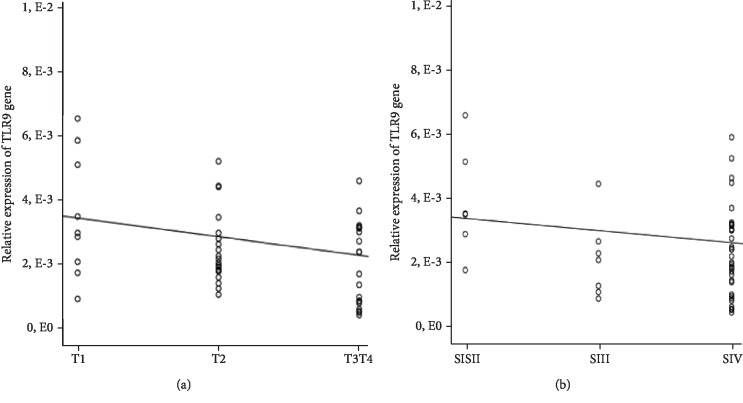
Relative expression of TLR9 mRNA according to (a) tumor size (*p* = 0.014, *r* = −0.314) and (b) clinical stage (*p* = 0.017, *r* = −0.177) (unilateral Spearman's correlation).

In this study, we also analyzed the relation between TLR9 mRNA expression level in PBMCs and clinicopathological characteristics of NPC. As shown in Table 5, TLR9 mRNA expression was significantly reduced in NPC patients with advanced state of the disease (patients with larger tumor size (T4)) (*p* = 0.013) and advanced clinical stage (SIII-SIV) (*p* = 0.0374) (Table 5) compared to patients with early state of the disease (T1, T2) (SI-SII), respectively. The transcriptomic analysis also showed that the relative expression of TLR9 mRNA was inversely correlated with the tumor size at diagnosis (*p* = 0.014; *r* = −0.314) but not with clinical stage (*p* = 0.117; *r* = −0.177) (Figure 2(a)).

## 4. Discussion

TLR9 is a PRR that is involved in the detection of intracellular unmethylated Cytosine-phosphate-Guanine (CpG) motives in DNA pathogen. This receptor is implicated in the activation of both innate and adaptive immune responses through the stimulation of various cell types, especially B cells and pDC cells. The capacity to respond properly to the TLR9 ligand may be damaged by single nucleotide polymorphisms (SNPs) within the TLR9 gene. They alter susceptibility to inflammatory diseases, infectious diseases, and cancers. However, in this study, we tried to find out if TLR9 promoter polymorphisms (TLR9-1486 T/C, TLR9-1237 T/C) were implicated in NPC physiopathology.

In this study, we demonstrated that the genetic variation in TLR9 gene might influence NPC progression (prognostic). Therefore, some significant associations were reported in our study between TLR9-1237 T/C (rs5743836) and clinicopathological parameters at the time of diagnosis, after treatment and survival rate of NPC patients. Our findings indicate that the -1237C mutated allele was associated with large tumor sizes at the time of diagnosis. This result can be explained under hypotheses based on the role of viral infection in the NPC pathogenesis and its close relationship with the TLR9 receptor, one of the TLR family that recognizes EBV DNA [32]. The endemic nature as well as carcinogenesis of NPC is considered a consequence of Epstein-Barr virus (EBV) infection, which is one major NPC etiological factor. The presence of viral proteins in tumor cells and NPC biopsies highlights the role of EBV in NPC development [33, 34]. Other than TLR9 stimulation, EBV may use TLR9 in order to escape from the host's immune surveillance [35]. During infection, EBV expresses BGLF5, EBV lytic-phase protein, which contributes to downregulating TLR9 levels through RNA degradation [36]. Fathallah et al. showed that the oncoprotein latent membrane protein 1 (LMP1) is a strong inhibitor of TLR9 transcription. However, overexpression of LMP1 reduces TLR9 promoter activity, mRNA, and protein levels [37]. Moreover, it has been demonstrated that the involvement of TLR9-MyD88 signaling by EBV inhibited TLR9 expression [38]. Therefore, we propose the hypothesis that, in NPC tumor cells, the presence of the mutated allele is associated with higher TLR9 mRNA expression levels. Since the viral proteins are present in NPC tumor cells, they can inhibit and degrade the TLR9 mRNA. Thus, EBV can escape the immune system from their hosts. Such a mechanism can suppress cancer immunity and promote tumor growth. Independent of EBV infection, it has been demonstrated that BL cells expressing TLR9-1237C allele are more resistant to apoptosis compared to cells expressing TLR9-1237T allele [39]. On the basis of these results, we may suppose that in NPC tumor cells, the presence of the TLR9-1237C allele can help tumor cells to escape apoptosis and enhance tumor growth.

In the present study, we also showed that after treatment, the same mutated TLR9-1237C allele was associated with both a better chance of complete remission and a protective effect against locoregional recurrence and distant metastasis. We have also proved that the mutated TLR9-1237CC genotype is associated with a better overall survival (OVS), distant metastasis-free survival (DMFS), and locoregional recurrence-free survival (LRRFS), among all NPC patients. These results seem contradictory to those reported here which show that the -1237C mutated allele was associated with large tumor size. These results can be explained under several hypotheses including both the presence of the TLR9-1237C mutated allele and the EBV viral load after treatment, just like the immunogenic role of radiotherapy. Our patients were recruited from the Department of Radiation Oncology. Patients with NPC are typically treated with radiation therapy (RT) rather than surgery because of the NPC anatomical limitations as well as high radiosensitivity. In our previous study, Hassen et al. showed that after treatment, the EBV viral load level declined significantly compared to that before treatment [6]. Recently, it has been revealed that the intensity-modulated radiotherapy (IMRT) reduces the number of DNA-EBV copies [40]. Such results may illustrate that treatment reduces the presence of EBV in NPC patients. Consequently, the EBV inhibitor effect on TLR9 was decreased. Thereafter, the TLR9 receptor was expressed after treatment. TLR9 is highly expressed in patients with the mutated TLR9-1237CC genotype compared to the other patients (with TT and TC genotypes) knowing that the presence of the TLR9-1237C mutated allele appears to stimulate the host immune response copies [22, 41]. Carvalho et al. reported that the C allele of the TLR9-1237 T/C polymorphism generates an IL 6-responding element. In this study, it was shown that in peripheral blood mononuclear cells (PBMCs) carrying the TLR9-1237C variant allele (with TC genotype), IL 6 upregulates TLR9 expression, which exacerbates cellular responses to CpG, including IL 6 production and B cell proliferation [22].

Otherwise, radiotherapy destroys cancer by emitting high rays on the cancer cells. This treatment kills cells by damaging their DNA and molecules that make up cancer cells. We think that such a process may lead to the release of EBV DNA in the host body. Furthermore, the stimulation of the immune system would result from activation of the TLR9 signaling. Thus, it has been demonstrated that TLR9-mediated detection of EBV DNA by pDCs leads to type I IFN (IFN-I) production. Such a process is involved in the simulation of the innate immune cells and promotes adaptive immune responses [42]. We may, therefore, conclude that the findings reported in this study would be due to the combined immunostimulant effects of radiation therapy and the presence TLR9-1237C allele which in turn increases TLR9 expression.

Several studies are interested in examining the effect of combining RT with TLR9 stimulation on antitumoral immunity, primary tumor growth retardation. Therefore, it has been demonstrated that CpG ODN 107 (TLR9 agonist) combined with irradiation (5 Gy) significantly suppresses the growth of glioma cell line in vitro. In vivo, this same study showed that the survival rate of mice was significantly increased by treatment with TLR9 agonist in combination with radiotherapy (10 Gy) compared with local radiotherapy alone in an orthotropic implantation model of nude mice. Such a combination therapy significantly decreases the microvessel density (MVD), VEGF level, and HIF-1 *α* expression [43]. Indeed, it has been demonstrated that TLR9 stimulation combined with RT treatment leads to humoral antitumor immune responses, increases tumoral infiltration, reduces pulmonary metastases, and improves the survival in mice bearing a murine lung adenocarcinoma (Lewis lung adenocarcinoma) [44]. Another study proved, in the animal models, that tumor therapeutic vaccine compound of TLR9 agonists (CpG ODN) and irradiated tumor cell tracking of two other CpG ODNs injections lead to a long-term antitumor immune response against aggressive tumors. Repeated vaccination improves experimental animals' survival compared with a single vaccination [45]. A similar combination therapy with radiotherapy and TLR stimulation was applied in patients with low-grade B cell lymphoma which develop specific CD4+ T cell response against a tumor [46]. It has been demonstrated that in non-small-cell lung cancer (NSCLC) cells, radiotherapy combined with TLR9 stimulation by the CpG ODN 7909 was able to downregulate PD-L1 (programmed death-ligand 1) expression which plays an important role in tumor immune escapes [47, 48]. All these results show that CpG ODN TLR9 stimulation is the potent enhancer of tumor response and as such has a potential to improve clinical radiotherapy bearing in mind that standard CpG oligonucleotides have been shown to be highly active in murine models while showing limited activity in humans.

## 5. Conclusion

The associations observed in this study suggest for the first time the involvement of functional promoter polymorphisms and mRNA expression of TLR9 gene in the NPC pathogenesis at the time of diagnosis, after radiotherapy treatment, and in NPC survival. Therefore, our results allowed us not only to better understand the impact of the TLR9 gene in NPC severity at the time of diagnosis but also to predict the role of the TLR9 gene in the progression of the disease after treatment.

## Figures and Tables

**Table 1:** Demographic and clinical characteristics of the study population.

Characteristics	Patients (*n* = 322)	
	*n*	*f* (%)
Mean age (mean ± SD)	42.62 ± 15.46	
Age at diagnosis (years)^a^		
≤35	104	32.3
>35	215	66.8
Sex^a^		
Women	100	31.1
Men	219	68
Tumor size^a^		
T1-T2	141	43.8
T3-T4	178	55.3
Lymph node status^a^		
N0	69	21.5
N+	250	77.6
Metastasis^a^		
M0	285	88.5
M+	21	6.5
Clinical stage^a^		
SI-SII	24	7.5
SIII-SIV	294	91.3
State after treatment^a^		
Complete remission (CR)	215	66.9
Locoregional recurrence (LRR)	32	9.9
Distant metastasis (DM)	31	9.6
Locoregional recurrence and distant metastasis (LRR+DM)	13	4
Death after treatment and after recurrence^a^		
No	252	78.3
Yes	39	12.1

*f*: frequencies; SD: standard deviation. ^a^The sum does not equal the total due to unavailable data.

**Table 2:** Promoter TLR9 polymorphisms and their associations with the clinicopathological parameters at diagnosis.

Clinicopathological parameters	TLR9-1486		TLR9-1237	
	TT	TC+CC	TT	TC+CC
Age at diagnosis (*n* = 319)				
≤35	35 (0.34)	69 (0.66)	74 (0.71)	30 (0.29)
>35	71 (0.33)	144 (0.67)	164 (0.76)	51 (0.24)
*p*		0.910		0.324
OR (CI 95%)	1	1.028 (0.62-1.68)	1	0.767 (0.45-1.30)
Sex (*n* = 319)				
Women	35 (0.35)	64 (0.65)	68 (0.69)	31 (0.31)
Men	71 (0.32)	149 (0.68)	169 (0.77)	51 (0.23)
*p*		0.588		0.124
OR (CI 95%)	1	1.147 (0.70-1.89)	1	0.662 (0.39-1.12)
Tumor size (*n* = 319)				
T1–T2	40 (0.28)	101 (0.72)	114 (0.81)	27 (0.19)
T3–T4	65 (0.36)	113 (0.64)	123 (0.69)	55 (0.31)
*p*		0.124		**0.017**
OR (CI 95%)	1	0.688 (0.43-1.11)	1	**1.888 (1.11-3.19)**
Lymph node status (*n* = 319)				
N0	19 (0.28)	50 (0.72)	54 (0.78)	15 (0.22)
N+	86 (0.34)	164 (0.66)	183 (0.73)	67 (0.27)
*p*		0.283		0.394
OR (CI 95%)	1	0.725 (0.40-1.30)	1	1.318 (0.69-2.49)
Metastasis (*n* = 306)				
M0	90 (0.32)	195 (0.68)	214 (0.75)	71 (0.25)
M+	8 (0.38)	13 (0.62)	17 (0.81)	4 (0.19)
*p*		0.536		0.792^a^
OR (CI 95%)	1	0.750 (0.30-1.87)	1	0.709 (0.23-2.17)
Clinical stage (*n* = 318)				
SI-SII	7 (0.29)	17 (0.71)	21 (0.88)	3 (0.12)
SIII-SIV	98 (0.33)	196 (0.67)	215 (0.73)	79 (0.27)
*p*		0.676		0.149^a^
OR (CI 95%)		0.824 (0.33-2.05)	1	2.572 (0.75-8.86)

^a^Fisher's exact test; OR: odds ratio; CI: confidence interval.

**Table 3:** Promoter TLR9 polymorphisms and their associations with the responses after treatment.

Responses after treatment parameters	TLR9-1486		TLR9-1237	
	TT	TC+CC	TT	TC+CC
Recurrence (*n* = 291)				
No	22 (0.29)	54 (0.71)	63 (0.83)	13 (0.17)
Yes	74 (0.34)	141 (0.66)	151 (0.70)	64 (0.30)
*p*		0.383		**0.031**
OR (CI 95%)	1	1.288 (0.73-2.28)	1	**0.486 (0.25-0.95)**
Locoregional recurrence (*n* = 260)				
No	74 (0.34)	141 (0.66)	151 (0.70)	64 (0.30)
Yes	13 (0.29)	32 (0.71)	38 (0.84)	7 (0.16)
*p*		0.474		**0.051**
OR (CI 95%)	1	1.292 (0.63-2.61)	1	**0.435 (0.18-1.02)**
Distant metastasis (*n* = 259)				
No	74 (34.4)	141 (65.6)	151 (70.2)	64 (29.8)
Yes	13 (29.6)	31 (70.5)	38 (86.4)	6 (13.6)
*p*		0.533		**0.028**
OR (CI 95%)	1	1.252 (0.62-2.53)	1	**0.373 (0.15-0.92)**
Death (*n* = 291)				
No	85 (0.34)	167 (0.66)	178 (0.71)	74 (0.29)
Yes	11 (0.23)	28 (0.78)	36 (0.92)	3 (0.08)
*p*		0.494		**0.003** ^**a**^
OR (CI 95%)	1	1.29 (0.62-2.73)	1	**0.20 (0.06-0.67)**

^a^Fisher's exact test; OR: odds ratio; CI: confidence interval.

**Table 4:** Univariate and multivariate Cox proportional hazards model for prognostic significance of pathologic features and *TLR9* promoter polymorphisms on overall survival (OVS), distant metastases-free survival (DMFS), and locoregional recurrence-free survival (LRRFS).

	Overall survival (OVS)						Distant metastases-free survival (DMFS)						Locoregional recurrence-free survival (LRRFS)					
	Univariate analysis			Multivariate analysis			Univariate analysis			Multivariate analysis			Univariate analysis			Multivariate analysis		
	HR	95% CI	*p* value	HR	95% CI	*p* value	HR	95% CI	*p* value	HR	95% CI	*p* value	HR	95% CI	*p* value	HR	95% CI	*p* value
Age^a^	4.622	1.80-11.83	0.001	4.05	1.57-10.4	0.004	2.540	1.22-5.28	0.013	ni			2.816	1.35-5.84	0.005	2.716	1.30-5.65	0.008
Sex^b^	2.322	1.06-5.05	0.034	ni			3.134	1.39-7.02	0.006	2.855	1.19-6.84	0.019	2.496	1.20-5.17	0.014	ni		
Tumor size^c^	0.657	0.35-1.23	0.190	ni			0.739	0.41-1.32	0.310	ni			0.991	0.55-1.78	0.979	ni		
Lymph node^d^	0.965	0.48-1.94	0.920	ni			1.269	0.26-2.56	0.507	ni			1.378	0.68-2.77	0.370	ni		
Metastasis^e^	3.708	1.44-9.54	0.007	3.35	1.29-8.69	0.013	5.264	2.42-11.42	0.00002	5.016	1.19-6.84	0.00007	1.130	0.27-4.67	0.866	ni		
Clinical stage^f^	0.910	0.323-2.56	0.858	ni			1.376	0.426-4.44	0.593	ni			1.496	0.46-4.82	0.500	ni		
TLR9-1486 T/C^g^	1.416	0.70-2.84	0.328	ns			1.308	0.68-2.50	0.417	ns			1.365	0.71-2.60	0.344	ns		
TLR9-1237 T/C^h^	0.215	0.06-0.697	0.010	0.244	0.75-0.798	0.02	0.414	0.17-0.98	0.045	0.388	0.15-1.00	0.048	0.468	0.20-1.04	0.065	0.507	0.22-1.13	0.099

HR: hazard ratio; ni: not included in multivariate analysis; ns: nonsignificant; a: ≤35 versus >35 years; b: men versus women; c: T1-T2 versus T3-T4; d: N0 versus N+; e: M0 versus M+; f: I–II versus III-IV; g, h: TT versus TC+CC.

**Table 5:** Relative expression of *TLR9* mRNA according to NPC clinicopathological parameters at diagnosis.

NPC clinicopathological parameters (*n*)	Median of TLR9 expression (*E*-03)	*p* value^a^
Age		0.696
Age ≤ 35 (17)	2.15	
Age > 35 (33)	2.24	
Sex		0.419
Women (14)	2.40	
Men (36)	2.01	
Tumor size		**0.013**
T1-T2-T3 (36)	2.52	
T4 (13)	1.35	
Lymph node		0.469
N0 (5)	3.13	
N+ (44)	2.11	
Metastasis		0.653
M0 (42)	2.20	
M+ (4)	1.94	
Clinical stage		**0.0374**
SI-SII (6)	3.46	
SIII-SIV (41)	2.03	

a: Mann-Whitney's test.

## Data Availability

The data used to support the findings of this study are available from the corresponding author upon request.
